# PPARβ/δ Regulates Glucocorticoid- and Sepsis-Induced FOXO1 Activation and Muscle Wasting

**DOI:** 10.1371/journal.pone.0059726

**Published:** 2013-03-21

**Authors:** Estibaliz Castillero, Nima Alamdari, Zaira Aversa, Aniket Gurav, Per-Olof Hasselgren

**Affiliations:** Department of Surgery, Beth Israel Deaconess Medical Center, Harvard Medical School, Boston, Massachusetts, United States of America; University of Kansas Medical Center, United States of America

## Abstract

FOXO1 is involved in glucocorticoid- and sepsis-induced muscle wasting, in part reflecting regulation of atrogin-1 and MuRF1. Mechanisms influencing FOXO1 expression in muscle wasting are poorly understood. We hypothesized that the transcription factor peroxisome proliferator-activated receptor β/δ (PPARβ/δ) upregulates muscle FOXO1 expression and activity with a downstream upregulation of atrogin-1 and MuRF1 expression during sepsis and glucocorticoid treatment and that inhibition of PPARβ/δ activity can prevent muscle wasting. We found that activation of PPARβ/δ in cultured myotubes increased FOXO1 activity, atrogin-1 and MuRF1 expression, protein degradation and myotube atrophy. Treatment of myotubes with dexamethasone increased PPARβ/δ expression and activity. Dexamethasone-induced FOXO1 activation and atrogin-1 and MuRF1 expression, protein degradation, and myotube atrophy were inhibited by PPARβ/δ blocker or siRNA. Importantly, muscle wasting induced in rats by dexamethasone or sepsis was prevented by treatment with a PPARβ/δ inhibitor. The present results suggest that PPARβ/δ regulates FOXO1 activation in glucocorticoid- and sepsis-induced muscle wasting and that treatment with a PPARβ/δ inhibitor may ameliorate loss of muscle mass in these conditions.

## Introduction

Muscle wasting caused by sepsis and high levels of glucocorticoids is characterized by increased expression of the ubiquitin ligases atrogin-1 and MuRF1 and stimulated ubiquitin-proteasome-dependent protein breakdown [1]–[3]. Atrogin-1 and MuRF1 were discovered approximately 10 years ago [4], [5] and are involved in the regulation of muscle mass in various catabolic conditions [6]. Their activity accounts for the specificity with regards to protein substrates that are ubiquitinated and degraded by the proteasome [4], [5]. Although the expression and activity of atrogin-1 and MuRF1 are regulated by multiple mechanisms [7], studies suggest that Forkhead box O (FOXO) transcription factors, in particular FOXO1, play a pivotal role in the regulation of atrogin-1 and MuRF1 expression in various muscle atrophy-related conditions, including sepsis and glucocorticoid treatment [3], [8]–[13]. FOXO-dependent gene activation can be regulated by increased overall expression of the transcription factors and by posttranslational modifications, including phosphorylation and acetylation [14]–[17]. The important role of FOXO transcription factors in the regulation of muscle mass is illustrated by their involvement not only in the regulation of atrogin-1 and MuRF1 expression and ubiquitin-proteasome-dependent proteolysis [10]–[13] but in the regulation of autophagy-lysosmal protein degradation as well [18], [19]. Understanding mechanisms regulating FOXO1 expression and activity during muscle wasting, therefore, has important clinical and translational implications.

Despite the important role of FOXO transcription factors in modulating muscle mass, the upstream regulation of the expression and activity of these transcription factors as well as their downstream influence on atrogin-1 and MuRF1 expression are not completely understood. In recent experiments, Nahle et al. [20] found evidence that FOXO1 expression and activity are regulated, at least in part, by the transcription factor PPARβ/δ. PPARβ/δ is a member of the PPAR transcription factor family [21], [22]. Members of this family participate in the regulation of genes involved in protein, carbohydrate, and lipid metabolism in multiple cell types and tissues [21]–[25]. In addition to PPARβ/δ, PPARα is also expressed in skeletal muscle where it is involved in the regulation of lipid metabolism [26]. In the study by Nahle et al. [20], fasting-induced upregulation of FOXO1 expression in heart muscle and diaphragm was blunted in PPARβ/δ -deficient mice. In addition, PPARβ/δ overexpression induced a robust increase in FOXO1 expression in cultured C2C12 muscle cells. Furthermore, analysis of the FOXO1 gene revealed PPAR response elements in the FOXO1 promoter region and overexpression of PPARβ/δ or pharmacological activation of PPARβ/δ with GW0742 transactivated the FOXO1 gene. In the same study [20], PPARβ/δ -induced activation of FOXO1 stimulated pyruvate dehydrogenase kinase 4 (PDK4) and suppressed glucose oxidation but other downstream targets of FOXO1 were not investigated. Although it is clear from the study by Nahle et al. [20] that FOXO1 expression is regulated by PPARβ/δ, it is not known if PPARβ/δ is involved in the regulation of atrogin-1, MuRF1, and muscle mass in catabolic conditions. This is important because atrogin-1 and MuRF1 are regulated by several factors in addition to FOXO transcription factors [7]. Importantly, it is also not known if inhibition of PPARβ/δ can prevent sepsis- and glucocorticoid-induced muscle wasting.

Some evidence suggesting a role of PPARβ/δ in the regulation of atrogin-1 and MuRF1 expression was reported by Constantin et al. [27]. In that study, treatment of rats with the PPARβ/δ agonist GW610742 resulted in increased atrogin-1 and MuRF1 mRNA and protein levels in soleus muscles. The interpretation of those results, however, is complicated for several reasons. First, no evidence of muscle wasting was found in the GW610742-treated rats. Muscle weight was not reported but body weight and muscle protein and DNA concentrations were not affected in the PPARβ/δ agonist-treated rats suggesting that muscle wasting was not induced in those experiments. Second, although muscle protein degradation rates were not determined, the 20S proteaosme chymotrypsin-like enzyme activity was unaffected by the PPARβ/δ agonist. Third, PPARβ/δ activity was not measured and it is not known if the treatment with GW610742 actually resulted in increased PPARβ/δ activity. Finally, studies were performed in the red, slow-twitch soleus muscle and the influence of GW610742 on atrogin-1 and MuRF1 expression in white, fast-twitch muscle was not investigated. This is important because the influence of sepsis, glucocorticoids, as well as other catabolic conditions, is particularly pronounced in fast-twitch skeletal muscle [28].

A study by Fuster et al. [29] provided additional evidence that PPARβ/δ may be involved in muscle wasting. In that study, PPARβ/δ mRNA levels were increased in skeletal muscle of tumor bearing rats, but although an association was found between increased expression of PPARβ/δ and loss of muscle mass, no mechanistic studies were performed testing the role of PPARβ/δ in cancer-induced muscle wasting. Also, the study by Fuster et al. [29] was focused on genes involved in fatty acid transport activation and oxidation and the relationship between PPARβ/δ and FOXO1, atrogin-1, and MuRF1 expression was not examined. In addition, it is not known from the study by Fuster et al. [29] if the increased mRNA levels for PPARβ/δ were associated with increased PPARβ/δ activity and whether inhibition of PPARβ/δ activity could rescue muscle from the catabolic effects of cancer cachexia.

Thus, the involvement of PPARβ/δ in muscle wasting and in the regulation of FOXO1, atrogin-1, and MuRF1 in catabolic muscle is incompletely understood at present. A better understanding of the role of PPARβ/δ in muscle wasting could have important clinical implications since evidence of PPARβ/δ -regulated expression of FOXO1, atrogin-1, and MuRF1 may make PPARβ/δ an important target for the prevention and treatment of muscle wasting. Here, we tested the hypotheses that FOXO1 activation and loss of muscle mass induced by glucocorticoids and sepsis is regulated by PPARβ/δ and that inhibition of PPARβ/δ activity can prevent muscle wasting in these conditions. Of note, although the potential relationship between PPARβ/δ and FOXO1, atrogin-1, and MuRF1 was examined in previous reports [20], [27], [29], the present study is important because it provides novel information about the mechanistic link between PPARβ/δ and sepsis- and glucocorticoid-induced muscle wasting as well as insight into the therapeutic implications of PPARβ/δ inhibition in these conditions.

## Materials and Methods

### Experiments in Cultured L6 Myotubes

L6 rat skeletal muscle cells (American Type Culture Collection, Manassas, VA, USA) were maintained and cultured as described in detail recently [30], [31] In short, L6 muscle cells were grown in Dulbeco’s Modified Eagle’s Medium (DMEM) supplemented with 10% fetal bovine serum (FBS), 100 U/ml penicillin, and 100 µg/ml streptomycin in 10% CO2 atmosphere at 37°C. When cells reached approximately 80% confluence, they were removed by trypsinization (0.25% trypsin in phosphate buffered saline, PBS) and seeded into 6- or 12-multiwell or 10-cm culture plates in the presence of 10% FBS until they reached approximately 70% confluence, at which time the medium was replaced with DMEM containing 2% FBS for induction of differentiation into myotubes. During this time (5–7 days), the myoblasts fused to form elongated, multinucleated myotubes. When myotubes had formed, cytosine arabinoside (10 µM) was added to the culture medium for 48 h in order to remove any remaining dividing myoblasts. In different experiments, myotubes were treated for 24 h with 0.1 µM of the PPARβ/δ agonist GW0742 (Sigma–Aldrich, St. Louis, MO, USA), 0.1 µM of the PPARβ/δ antagonist GSK0660 (Tocris Bioscience, Ellisville, MI, USA), 1 µM dexamethasone (Sigma–Aldrich), or 10 µM of RU38486 (Sigma-Aldrich). Control myotubes were treated with solvent (0.1% DMSO for GW0742 and 0.1% ethanol for GSK0660, dexamethasone, and RU38486).

The concentration of GW0742 used here was based on a previous report in which the drug activated PPARβ/δ-dependent gene transcription in cultured muscle cells in a dose-dependent manner with concentrations ranging from 0.04 to 0.12 µM [20]. In previous reports, GSK0660 was used in cell culture experiments at a concentration of 1 µM [32], [33]. Because in our experiments, GSK0660 at 1 µM showed evidence of cell toxicity (cell rounding and detachment and activated protein breakdown), we tested the effects of lower GSK0660 concentrations. Results from those experiments showed that GSK0660 at a concentration of 0.1 µM significantly reduced protein degradation in control and dexamethasone-treated myotubes whereas 0.05 µM GSK0660 did not influence myotube protein degradation. A GSK0660 concentration of 0.1 µM was therefore used in our experiments; this concentration was validated by its inhibition of GW0742-induced activation of PPARβ/δ in cultured myotubes (see Results).

The concentrations of dexamethasone and RU38486 used here were based on previous reports from our laboratory in which these drugs stimulated and prevented dexamethasone-induced protein degradation, respectively, in a dose-dependent manner in cultured L6 myotubes [30], [31], [34].

In other experiments, differentiated L6 myotubes were transfected with PPARβ/δ or FOXO1 siRNA or corresponding concentrations of non-targeting (scrambled) RNA (Dharmacon Technologies, Lake Placid, NY, USA). The RNA constructs were added to the culture medium at a concentration of 165 nM together with the transfection reagent Lipofectamine RNAiMAX (Invitrogen, Grand Island, NY, USA). After 5 h, the medium was changed and the myotubes were cultured for an additional 48 h in DMEM with 2% FBS in order to allow for continued gene silencing. In the PPARβ/δ silencing experiment, myotubes were then treated for 24 h with 1 µM dexamethasone or solvent (0.1% ethanol). In the FOXO1 silencing experiment, myotubes were treated for 24 h with 0.1 µM GW0742 or 0.1% DMSO.

After the different treatments, myotube morphology and diameter and protein degradation rates were determined as described in detail [30], [31]. In other experiments, mRNA and protein levels for FOXO1, atrogin-1, MuRF1, and PPARβ/δ were determined by real-time PCR and Western blot, respectively (see below). PPARβ/δ and FOXO1 activity were determined as described below.

### Experiments in Dexamethasone-treated and Septic Rats

Two series of experiments were performed in male Sprague-Dawley rats (50–60 g bw) to determine the effect of GSK0660 on muscle wasting. In the first series, rats were treated with dexamethasone (10 mg/kg ip) or corresponding volume of vehicle. This protocol for dexamethasone treatment of rats was described in detail previously and results in increased ubiquitin-proteasome-dependent muscle proteolysis and increased expression of FOXO1, atrogin-1, and MuRF1 [12], [35]–[37]. In the second series of in vivo experiments, sepsis was induced by cecal ligation and puncture (CLP) as described in detail previously [12], [35]–[39]. Other rats were sham-operated, i.e., they underwent laparotomy and manipulation, but no ligation or puncture of the cecum. The septic model used here was used in several previous studies from our laboratory and results in a reproducible increase in muscle proteolysis, expression of FOXO1, atrogin-1, and MuRF1 and loss of muscle mass [12], [35]–[39]. The model is clinically relevant because it resembles the situation in patients presenting with septic peritonitis caused by intraabdominal abscesses and devitalized tissue. Of note, small growing rats were used in the present study. Rats of the same size were used in several previous reports in which we studied sepsis- and glucocorticoid-induced muscle wasting because they have lower extremity muscles that are thin enough to allow for in vitro incubation and measurement of protein turnover rates under physiological conditions [28], [35], [38]. Rats of the same size were used here to make it possible to compare the present observations with previous studies. Importantly, we reported previously that metabolic changes in skeletal muscle during sepsis induced by CLP were similar in small growing rats and adult rats [40]. Several metabolic and molecular changes seen in muscle after CLP in young growing rats are present in muscle from adult septic patients [41].

Groups of rats were treated with GSK0660 (1 mg/kg ip) or corresponding volume of vehicle (0.5 ml of 50% ethanol ip) 2 h before treatment with dexamethasone or induction of sepsis. This dose of GSK0660 was based on a previous report by Sanchez-Siles et al [42] and did not induce toxic side-effects in the present study (see below in Results). In fact, a dose as high as 10 mg/kg was reported to be safe in mice [42]. Sixteen hours after dexamethasone treatment or CLP, extensor digitorum longus (EDL) muscles were harvested, immediately frozen in liquid nitrogen and stored at −80°C until analyses were performed as described below. EDL muscles were studied here because white, fast-twitch skeletal muscle is particularly sensitive to the effects of catabolic conditions, including glucocorticoid treatment and sepsis [28], [35]. Blood was drawn by heart puncture for measurement of serum concentrations of the liver enzymes alanine aminotransferase (ALT) and aspartate aminotransferase (AST) using commercial activity assay kits (Biovision, Mountain View, CA, USA).

Of note, the in vitro and in vivo experimental models used in the present study, i.e., dexamethasone-treated myotubes and rats and sepsis in rats induced by CLP, were employed in multiple previous reports from our laboratory [12], [28], [30], [31], [34]–[41]. In those studies, dexamethasone treatment of myotubes or rats and sepsis in rats resulted in increased protein degradation, loss of muscle mass, and myotube atrophy. In another study from our laboratory, electron microscopy provided evidence of sarcomere disruption, disintegration of Z-disks, and morphological changes of mitochondria (including swelling and loss of membrane structures) in skeletal muscle from septic rats [43]. In order to provide further characterization of our experimental septic model, we performed an additional study in muscles from sham-operated and septic rats for morphological analysis. In that experiment, tibialis anterior muscles (a white fast-twitch skeletal muscle similar to the EDL muscle) were harvested 16 h after sham-operation or CLP. The muscles were frozen in pre-cooled isopentane and stored at −80°C until analysis. Microscopic sections were prepared from the mid portion of each muscle, frozen sections were immunostained with laminin, and digital photographs were taken. The cross sectional area (CSA) was measure for a minimum of 50 fibers per muscle with one muscle each from 6 sham-operated and 6 septic rats. Result from this experiment showed histological evidence of muscle atrophy with the muscle fiber CSA in septic rats being 65±12% of the CSA in muscles from sham-operated rats (p<0.05). Taken together, the observations from our previous reports and the new results of decreased CSA in muscle from septic rats provide biochemical, molecular, and histological evidence that both our in vitro and in vivo models used here are associated with increased proteolysis and atrophy.

### Ethics Statement

All animal experiments were carried out in strict accordance with the recommendations in the Guide for the Care and Use of Laboratory Animals and the National Institutes of Health. The animal protocols were approved by the Institutional Animal Care and Use Committee at the Beth Israel Deaconess Medical Center, Harvard Medical School, Boston, MA, USA. All surgeries were performed under sodium pentobarbital anesthesia, and all efforts were made to minimize suffering.

### Preparation of Total and Nuclear Cell and Muscle Extracts

Total cell and muscle lysates were prepared by homogenizing the muscles and harvesting the myotubes directly in RIPA buffer (50 mM Tris–HCl, 150 mM NaCl, 0.5% sodium deoxycholate, 0.1% SDS, and 1% Nonidet P-40) containing Protease Inhibitor Cocktail Tablets (Roche Applied Science, Indianapolis, IN, USA). Myotube extracts were briefly sonicated using a Model 100 Sonic Dismembrator (Fisher Scientific, Asheville, NC, USA). Cell and muscle extracts were centrifuged at 14,000 *g* for 10 min at 4°C. Nuclear extracts were prepared using the NE-PER® Nuclear and Cytoplasmic Extraction Reagents (Thermo Fisher Scientific) according to the manufacturer’s instructions. Concentrations of soluble proteins in the lysates were determined by using the Bradford Protein Assay Kit (Thermo Fisher Scientific) with bovine serum albumin as standard. Nuclear extracts and cell and muscle lysates were stored at −80°C until analyzed.

### FOXO1 Activity Determination

FOXO1 DNA binding activity was determined in L6 myotube nuclear extracts using the Trans AM FKHR Transcription Factor Assay Kit (Active Motif, Carlsbad, CA, USA). In this assay, a forkhead box element immobilized onto a 96-well plate is combined with the nuclear extracts. FOXO transcription factors contained in the extracts bind to the DNA molecule, and FOXO1 is detected by a specific primary antibody. A horseradish peroxidase-conjugated secondary antibody is then used for colorimetric detection in a spectrophotometric plate reader.

### PPARβ/δ Activity Determination

PPARβ/δ DNA binding activity was determined in L6 myotube and EDL nuclear extracts using the PPARδ Transcription Factor Assay Kit (Cayman, Ann Arbor, MI, USA). In this assay, a double stranded consensus sequence containing the peroxisome proliferator response element (PPRE) immobilized onto a 96-well plate is combined with the nuclear extracts. PPARs contained in the extracts bind specifically to the PPREs, and PPARβ/δ is detected by using a specific primary antibody. A horseradish peroxidase-conjugated secondary antibody is then used for colorimetric detection in a spectrophotometric plate reader.

### Real-time PCR

Messenger RNA levels for FOXO1, atrogin-1, MuRF1, PPARβ/δ, and PPARα were determined by real-time PCR performed as described in detail in recent reports from this laboratory [12], [30], [31], [36], [37]. Multiplex RT-PCR with amplification of 18S RNA as endogenous control, TaqMan analysis and subsequent calculations were performed with an ABI Prism 7700 Sequence Detection System (Perkin Elmer, Foster City, CA, USA). The sequences of the forward, reverse, and double-labeled oligonucleotides for FOXO1, atrogin-1 and MuRF1 used here were reported recently [12], [30], [31], [37]. PPARβ/δ and PPARα mRNA levels were determined using the ABI TaqMan Gene Expression Assay (Assay ID: Rn01441087_m1 for PPARβ/δ and Assay ID: Rn00566193_m1 for PPARα). Amplification of 18S RNA was performed in the same reaction tubes as an internal standard with an alternatively labeled probe (VIC-labeled probe) to distinguish its product from those derived from FOXO1, atrogin-1, MuRF1, PPARβ/δ and PPARα mRNA. Atrogin-1, MuRF1, FOXO1, PPARβ/δ and PPARα mRNA concentrations were normalized to the 18S mRNA levels and were expressed as arbitrary units (AU).

### Western Blotting

Western blotting was performed to determine protein expression of total FOXO1, p-FOXO1(Ser 256), atrogin-1, MuRF-1, PPARβ/δ, and the glucocorticoid receptor (GR). The protocol used for Western blotting was described in detail [12], [30], [31], [36], [37]. The following primary antibodies and the appropriate secondary antibodies were used: a rabbit polyclonal anti-rat FOXO1 antibody (1∶1000, Cell Signaling Technology, Danver, MA, USA), a rabbit polyclonal anti-rat phospho (Ser 256)-FOXO1 antibody (1∶1000, Cell Signaling Technology), a goat anti-human PPARβ/δ antibody (1∶1000, Novus Biologicals, Littleton, CO, USA), a rabbit polyclonal anti-mouse atrogin-1 antibody (1∶1000; kindly supplied by Dr. Stewart Lecker, Harvard Medical School, Boston, MA, USA), a mouse polyclonal anti-rat MuRF1 antibody (1∶1000; kindly supplied by Regeneron Pharmaceuticals, NY, USA), and a rabbit anti-human GR antibody (1∶1000, Santa Cruz Biotechnology, Santa Cruz, CA, USA). A mouse monoclonal anti-rat α tubulin antibody (1∶5000, Sigma–Aldrich, St. Louis, MO, USA) was used for loading control when total muscle extracts were immunoblotted and an anti-rat lamin A/C antibody (1∶2000, Cell Signaling Technology) was used for loading control when nuclear extracts were analyzed. Immunoreactive protein bands were detected by using the Western Lightning kit for enhanced chemiluminescence detection (PerkinElmer Life Sciences) and analyzed using the public domain Image J program (http://rsb.info.nih.gov.ezp-prod1.hul.harvard.edu/ij/index.html). The bands were quantified by densitometry and normalized to the appropriate loading controls.

### Co-immunoprecipitation

Co-immunoprecipitation was performed in order to examine whether there is a physical interaction between PPARβ/δ and the GR. Whole cell lysate proteins from myotubes treated with dexamethasone or solvent were extracted using Pierce IP Lysis Buffer (Thermo Scientific, Rockford, IL, USA). This moderate strength modified RIPA buffer effectively solubilizes cellular proteins but does not disrupt protein complexes. Protein extracts (50 µg) were immunoprecipitated with a goat anti-human PPARβ/δ antibody (1∶1000, Novus Biologicals, Littleton, CO, USA) using the Catch and Release v2.0 Reversible Immunoprecipitation System (Millipore, Billerica, MA, USA) following the manufacturer’s instructions. Samples were mixed in the spin columns with the antibody overnight at 4°C. The columns were then washed three times with wash buffer, and bound proteins were eluted with 2× SDS sample buffer at 95°C for 5 min and were subjected to SDS-PAGE using 7.5% gels, followed by transfer to PVDF membranes. The membranes were blocked with 5% non-fat milk in TTBS buffer and incubated with the following primary antibodies and the appropriate secondary antibodies: a rabbit anti-human PPARβ/δ antibody (1∶1000, Santa Cruz Biotechnology) and a rabbit anti-human GR antibody (1∶1000, Santa Cruz Biotechnology).

In other experiments, levels of acetylated FOXO1 (Ac-FOXO1) were determined in cultured myotubes or rat EDL muscle by co-immunoprecipitation performed as described in detail recently [44]. In short, nuclear proteins (300 µg) were pulled down with an anti-acetyl lysine antibody (Cell Signaling Technology) followed by Western blotting using a rabbit polyclonal anti-rat FOXO1 antibody (Cell Signaling Technology).

When Western blotting was performed (alone or as part of co-immunoprecipitation), representative blots are shown in the upper panel of each figure with data from quantification shown below as bars. The blots correspond to the groups shown in the bars and are shown in the same order as the bars.

### Statistics

Results are reported as means ± SEM. Statistical analysis was performed by using Student’s *t*-test or one-way ANOVA followed by Tukey’s post hoc test as appropriate. p<0.05 was considered statistically significant.

## Results

### Activation of PPARβ/δ Results in FOXO1-dependent Upregulation of Atrogin-1 and MuRF1 in Cultured Myotubes

**Figure 1:**
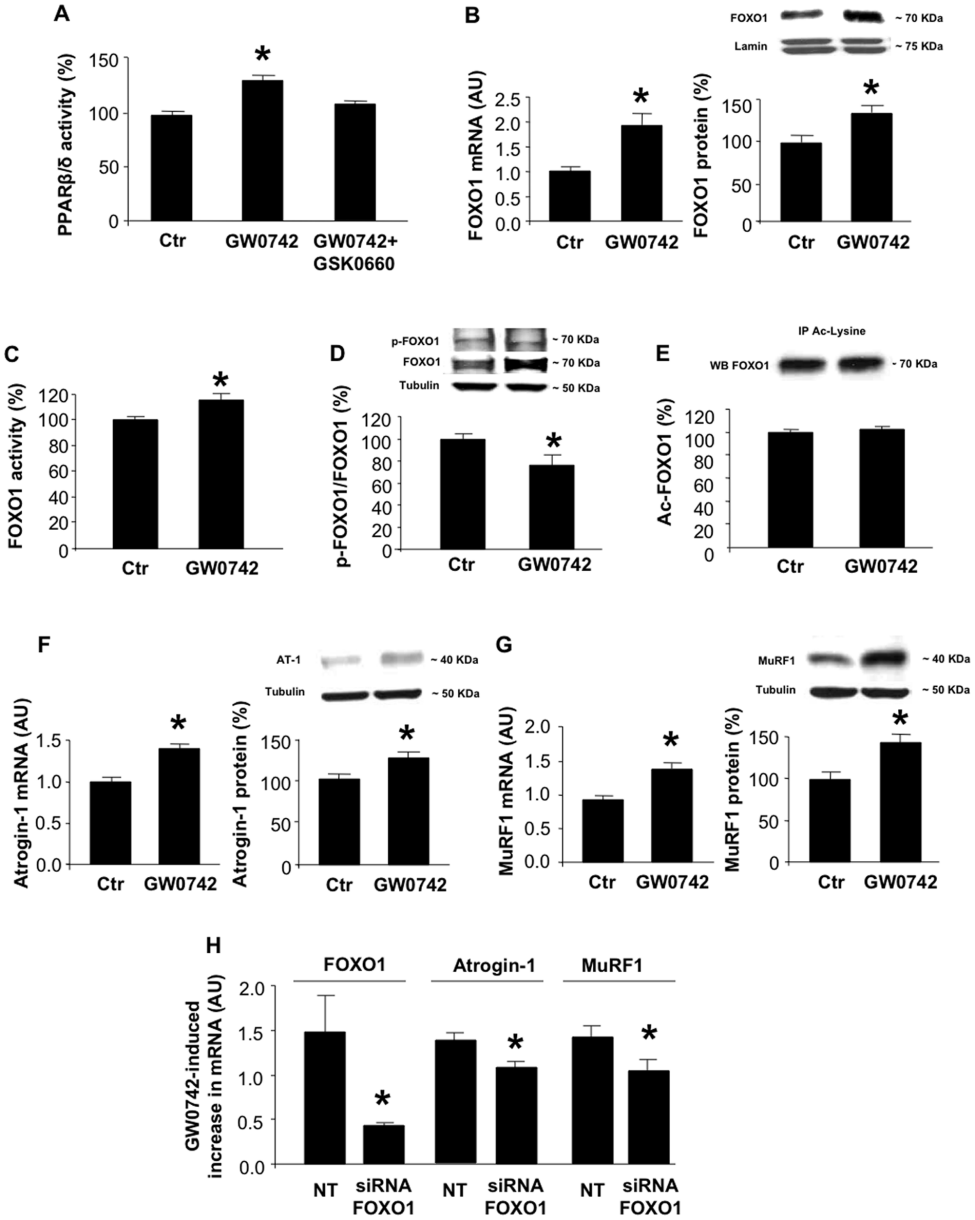
PPARβ/δ activation upregulates atrogin-1 and MuRF1 expression through a FOXO1-dependent mechanism in L6 myotubes. *A*) PPARβ/δ activity in cultured L6 myotubes treated for 24 h with 0.1 µM GW0742 in the absence or presence of 0.1 µM GSK0660. Results are expressed as percent of control (Ctr). *B*) FOXO1 mRNA (left panel) and protein levels (right panel) in cultured L6 myotubes after treatment for 24 h with 0.1 µM GW0742. Representative Western blots are shown in the upper and densitometric quantifications in the lower panel. A/C lamin was determined for loading control. *C*) FOXO1 DNA binding activity in control and GW0742-treated myotubes. *D*) p-FOXO1(Ser 256) and total FOXO1 protein levels (upper panel) and calculated p-FOXO1/FOXO1 ratio in cultured L6 myotubes after treatment for 24 h with 0.1 µM GW0742. The calculated p-FOXO1/FOXO1 ratio was based on densitometric quantifications of p-FOXO1 and total FOXO1 levels on Western blots. *E*) Ac-FOXO1 levels in control and GW0742-treated myotubes determined by co-immunoprecipitation. *F)* Atrogin-1 mRNA and protein levels in cultured L6 myotubes after treatment for 24 h with 0.1 µM GW0742 and in control myotubes. α-Tubulin was determined for loading control. *G*) MuRF1 mRNA and protein levels in control and GW0742-treated myotubes. *H*) GW0742-induced increase in mRNA levels for FOXO1, atrogin-1, and MuRF1 in myotubes transfected with a non-targeting (NT) or FOXO1 siRNA. Results are means ± SEM with n = 7 or 8/group. *p<0.05 vs control (Ctr) or NT groups by Student’s t-test (panel B-H) or ANOVA (panel A).

In initial experiments, we tested the effects of the PPARβ/δ agonist GW0742 [45] on the expression of FOXO1, atrogin-1 and MuRF1 in cultured L6 myotubes. GW0742 was found in a recent study to transactivate the FOXO1 promoter in cultured C2C12 myoblasts but PPARβ/δ activity was not measured in those experiments [20]. Here, we found that treatment of L6 myotubes with GW0742 resulted in an approximately 30% increase in PPARβ/δ activity (Figure 1A). This effect of GW0742 was blocked by the PPARβ/δ inhibitor GSK0660 [46], supporting the specificity of GW0742 as a PPARβ/δ activator. When myotubes were treated with GW0742, mRNA and protein levels for FOXO1 increased (Figure 1B) and the increased FOXO1 expression was accompanied by stimulated FOXO1 DNA-binding activity (Figure 1C). Although reduced phosphorylation is an important mechanism of activation of FOXO transcription factors [47], recent studies suggest that in certain catabolic conditions, such as starvation [8] and sepsis [12], FOXO1 activation is caused by increased levels of total FOXO1, resulting in reduced p-FOXO1/FOXO1 ratio (even in the presence of increased p-FOXO1 levels). To test if a similar mechanism is involved in GW0742-induced activation of FOXO1, cellular levels of p-FOXO1(Ser 256) and total FOXO1 were determined. Treatment of the myotubes with GW0742 resulted in a reduced p-FOXO1/FOXO1 ratio reflecting a more pronounced increase in total FOXO1 than in p-FOXO1 levels (Figure 1D). Thus, the increased FOXO1 activity in GW0742-treated myotubes probably reflected a net increase in unphosphorylated (activated) FOXO1.

In addition to phosphorylation, acetylation may also influence FOXO1 activity [44], [48]–[50]. We next tested whether GW0742-induced PPARβ/δ activation may be associated with increased acetylation of FOXO1 by measuring myotube levels of Ac-FOXO1. Treatment of myotubes with GW0742 did not influence Ac-FOXO1 levels (Figure 1E) suggesting that the GW0742-induced FOXO1 activation was not regulated by increased FOXO1 acetylation under the present experimental conditions.

We next examined whether GW0742 would increase the expression of atrogin-1 and MuRF1. Treatment of the myotubes with GW0742 resulted in increased mRNA and protein levels for both atrogin-1 (Figure 1F) and MuRF1 (Figure 1G). To test the link between increased expression and activity of FOXO1 and atrogin-1 and MuRF1 expression in GW0742-treated myotubes, we employed FOXO1 siRNA. Transfection of the myotubes with FOXO1 siRNA resulted in a robust reduction of the GW0742-induced increase in FOXO1 expression and a significant (albeit smaller) inhibition of the GW0742-induced increase in atrogin-1 and MuRF1 expression, suggesting that atrogin-1 and MuRF1 expression in PPARβ/δ-activated muscle cells is at least in part regulated by FOXO1 (Figure 1H).

### Treatment of Cultured Muscle Cells with the PPARβ/δ Agonist GW0742 Increases Protein Degradation and Induces Myotube Atrophy

**Figure 2:**
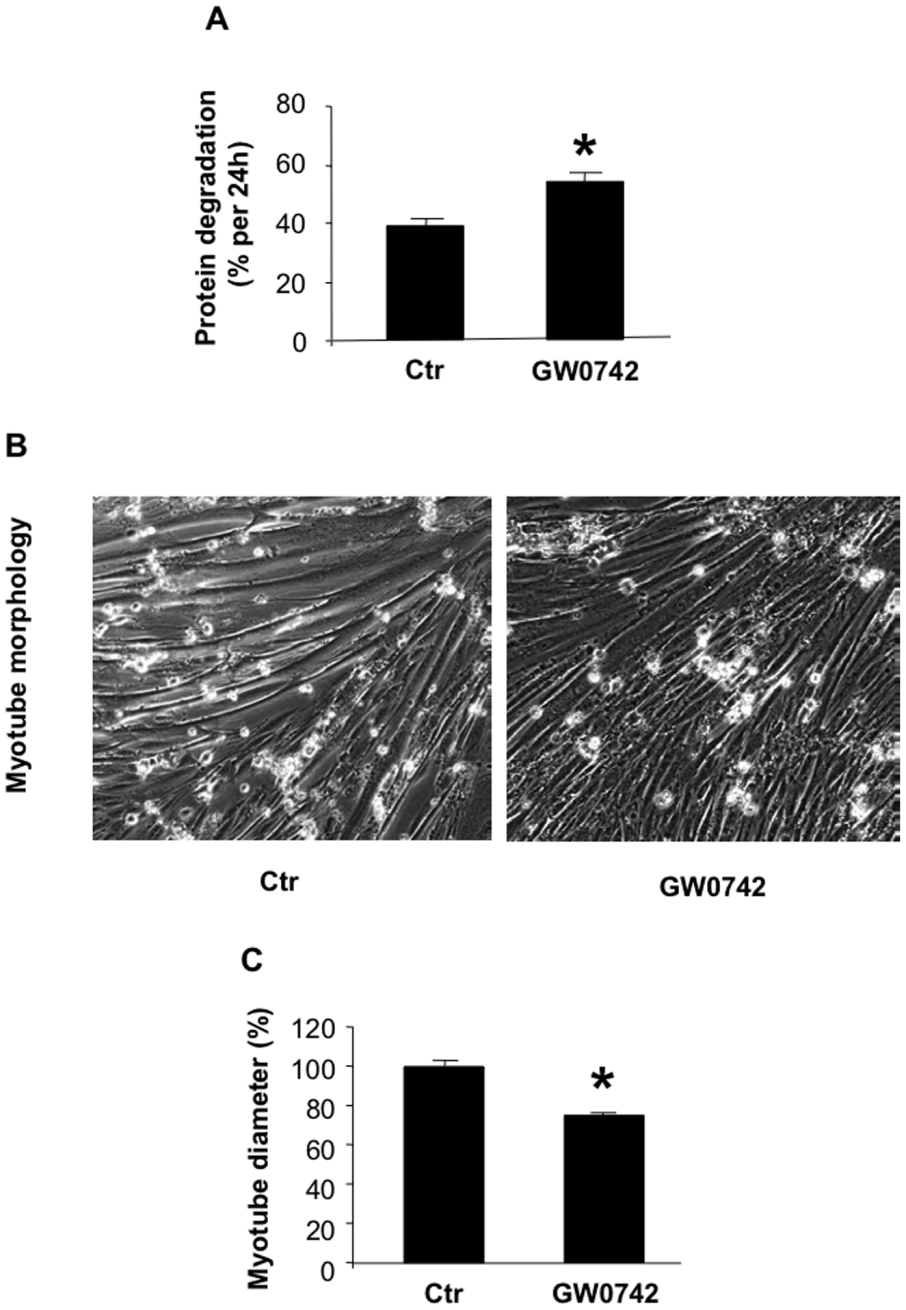
The PPARβ/δ agonist GW0742 increases protein degradation and induces atrophy of cultured L6 myotubes. *A*) Protein degradation (% per 24 h) in myotubes treated for 24 h with 0.1 µM GW0742 or vehicle (Ctr). *B*) Morphology of cultured L6 myotubes after treatment for 24 h with 0.1 µM GW0742 or vehicle (Ctr). Myotubes were photographed under a phase contrast microscope at 100× magnification. *C*) Diameter of cultured myotubes after treatment for 24 h with 0.1 µM GW0742 or vehicle (Ctr). Results are means ± SEM with n = 8/group.*p<0.05 vs control.

Importantly, the GW0742-induced PPARβ/δ activation was accompanied by an approximately 35% increase in myotube protein degradation (Figure 2A) and a 30% reduction of myotube diameter (Figure 2B and C). Together, results shown in Figure 1 and 2 suggest that GW0742 activates PPARβ/δ in skeletal muscle and that this activation results in upregulated expression and activity of FOXO1 and FOXO1-dependent expression of atrogin-1 and MuRF1. In addition, results suggest that these effects of PPARβ/δ activation are accompanied by stimulated muscle proteolysis and myotube atrophy.

### Treatment of Cultured Myotubes with Dexamethasone Increases PPARβ/δ Activity through a Glucocorticoid Receptor (GR)-dependent Mechanism

**Figure 3:**
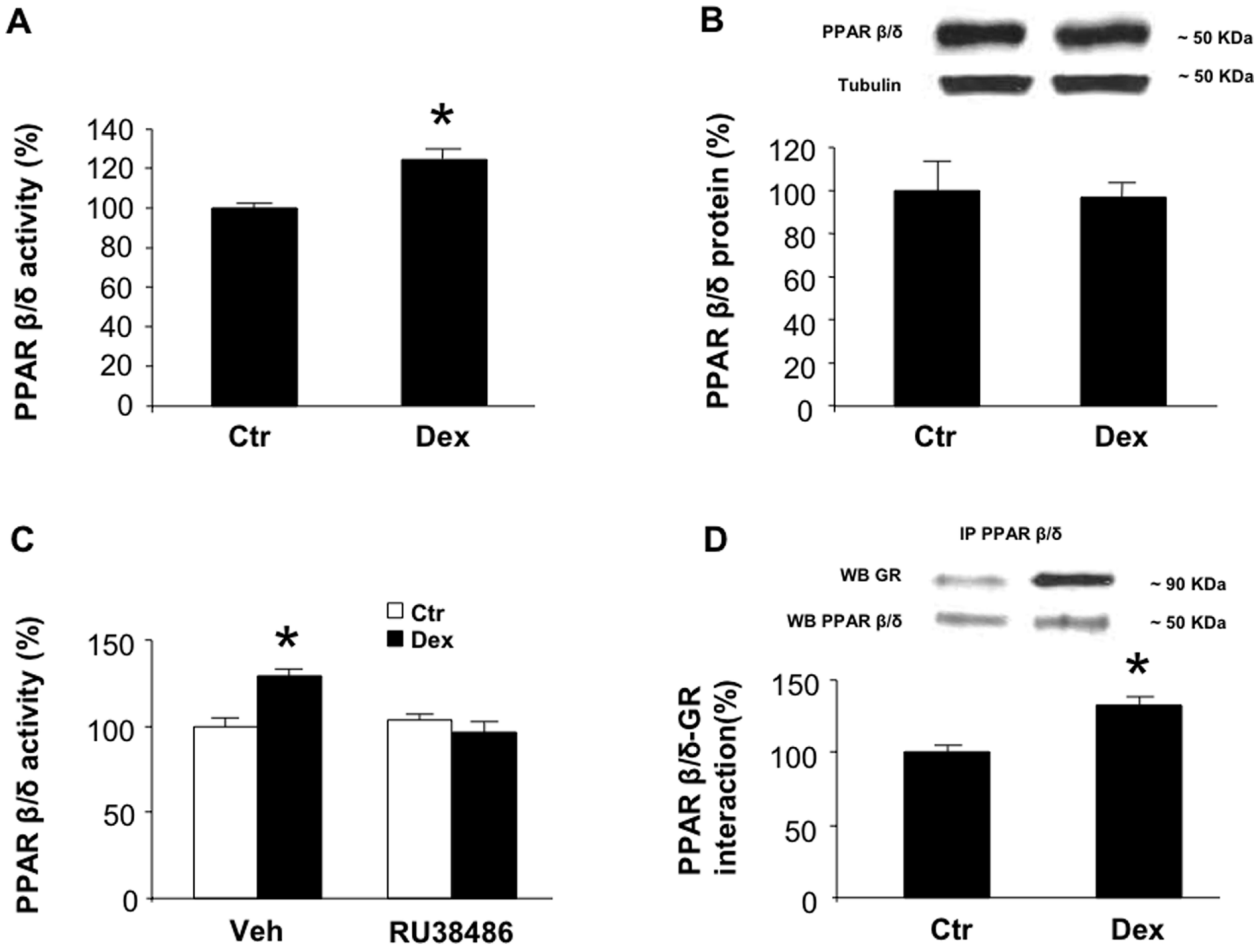
Dexamethasone increases PPARβ/δ activity in cultured L6 myotubes through a GR-dependent mechanism. *A*) PPARβ/δ activity in myotubes treated for 24 h with 1 µM dexamethasone or vehicle (Ctr). Results are expressed as percent of control. *B*) PPARβ/δ protein levels determined by Western blotting in total cell lysates from control and dexamethasone-treated myotubes. Representative Western blots are shown in the upper panel and densitometric quantifications in the lower panel. α-Tubulin was determined for loading control. *C*) PPARβ/δ activity in control and dexamethasone-treated myotubes. Myotubes were treated for 24 h with dexamethasone (1 µM) in the absence or presence of 10 µM RU38486. *D*) PPARβ/δ-GR interaction determined by co-immunoprecipitation of myotube proteins extracted from whole cell lysates after treatment of the myotubes for 24 h with 1 µM dexamethasone or vehicle (Ctr). A PPARβ/δ antibody was used for pull-down followed by immuno blotting with a GR or PPARβ/δ antibody. Representative immunoblots are shown in the upper panel and densitometric quantifications of the upper bands in the immunoblts are shown in the lower panel. Results were calculated as percent of control and are means ± SEM with n = 7 or 8/group. *p<0.05 vs Ctr by Student’s t-test (panel A and D) or ANOVA (panel C).

In previous studies, treatment of cultured muscle cells with dexamethasone resulted in upregulated expression of atrogin-1 and MuRF1, increased protein degradation, and atrophy [30], [31]. Those were important observations because sepsis-induced muscle wasting is regulated by glucocorticoids [35], [38] and high levels of glucocorticoids by themselves stimulate muscle proteolysis [2], [3]. Thus, dexamethasone-treated cultured myotubes provide a relevant in vitro model of muscle wasting [31]. Previous reports suggest that glucocorticoids upregulate the expression and activity of PPARα and γ [51]–[54] but the influence of glucocorticoids on PPARβ/δ expression and activity is not known. Here, we found that treatment of cultured L6 myotubes with dexamethasone resulted in an approximately 20% increase in PPARβ/δ activity (Figure 3A). This activation did not reflect increased abundance of PPARβ/δ since PPARβ/δ protein levels were unchanged in dexamethasone-treated myotubes (Figure 3B). Previous studies suggest that glucocorticoid-induced activation of PPARγ reflects an interaction between PPARγ and the GR [54]. It is not known if the GR plays a similar role in PPARβ/δ activation. We therefore tested the role of the GR in dexamethasone-induced PPARβ/δ activation in cultured L6 myotubes. When myotubes were treated with the GR antagonist RU38486 [55], the dexamethasone-induced activation of PPARβ/δ was blocked (Figure 3C). Co-immunoprecipitation provided evidence for increased interaction between PPARβ/δ and the GR in dexamethasone-treated myotubes (Figure 3D). Together, results shown in Figure 3 suggest that glucocorticoids activate PPARβ/δ in skeletal muscle cells and that this effect of glucocorticoids is at least in part regulated by the GR, and is associated with protein-protein interaction between PPARβ/δ and the GR.

### The PPARβ/δ Antagonist GSK0660 Blocks the Catabolic Effects of Dexamethasone in Cultured Myotubes

**Figure 4:**
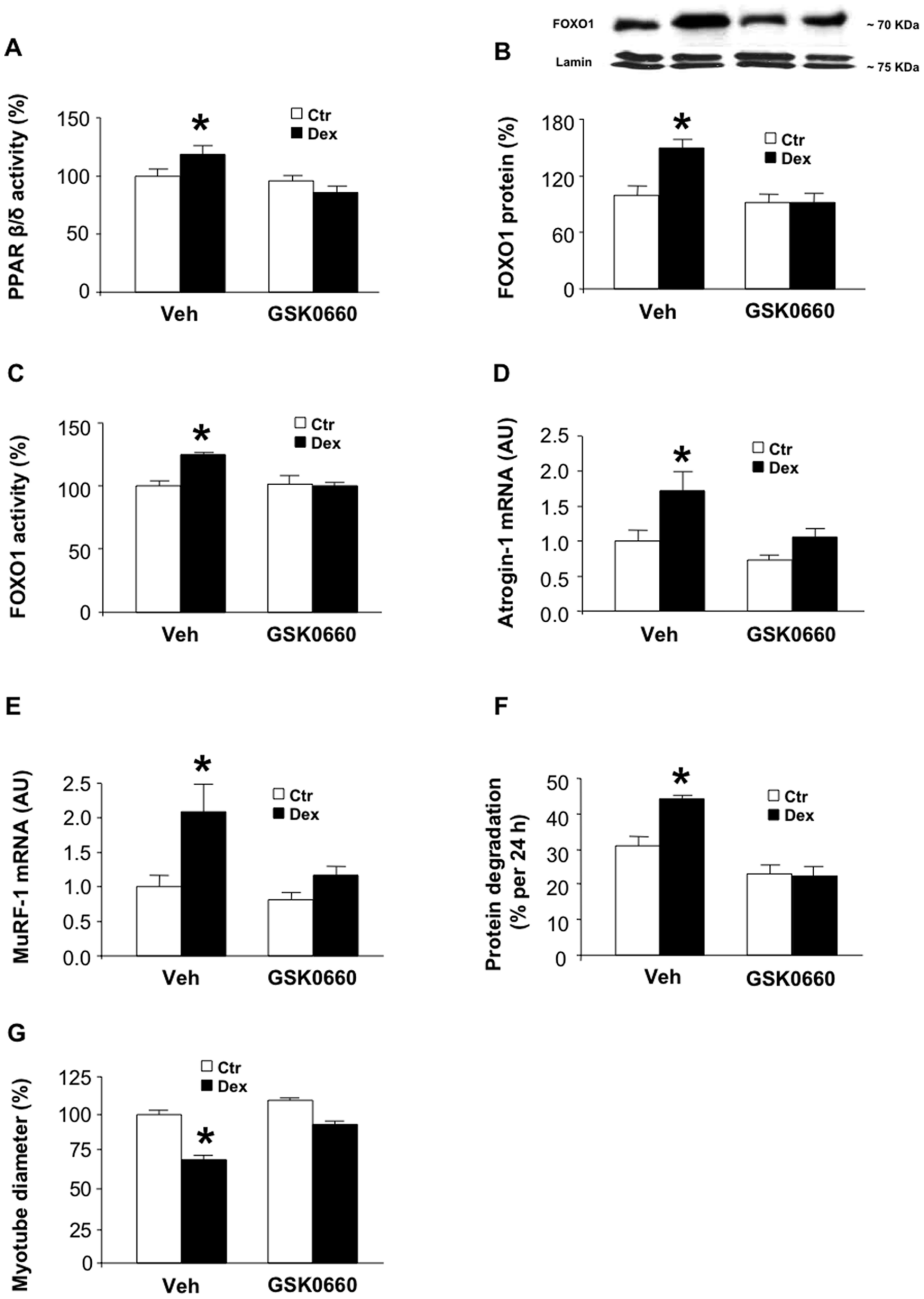
The PPARβ/δ antagonist GSK0660 blocks the catabolic effects of dexamethasone in cultured L6 myotubes. *A*) PPARβ/δ activity in myotubes treated with or without 1 µM dexamethasone in the absence or presence of 0.1 µM GSK0660. *B*) FOXO1 protein levels in myotubes treated as described in panel A. FOXO1 levels were determined by Western blotting of nuclear extracts and A/C lamin levels were determined as loading controls. *C*) FOXO1 activity in myotubes treated for 24 h with 1 µM dexamethasone in the absence or presence of 0.1 µM GSK0660. *D*) Atrogin-1 and *E*) MuRF1 mRNA levels in myotubes treated for 24 h with or without 1 µM dexamethasone in the absence or presence of 0.1 µM GSK0660. *F*) Protein degradation (% per 24 h) in myotubes treated for 24 h with or without 1 µM dexamethasone in the absence or presence of 0.1 µM GSK0660. *G*) Diameter of myotubes after treatment for 24 h with or without 1 µM dexamethasone in the absence or presence of 0.1 µM GSK0660. Results are means ± SEM with n = 7 or 8/group. *p<0.05 vs Ctr/Veh by ANOVA.

Having established that PPARβ/δ is activated by glucocorticoids and that PPARβ/δ can upregulate the expression and activity of FOXO1 as well as expression of atrogin-1 and MuRF1, stimulate protein degradation, and cause muscle cell atrophy, we next tested whether dexamethasone-induced activation of FOXO1, expression of atrogin-1 and MuRF1 and protein degradation can be inhibited by blocking PPARβ/δ activity. Two approaches were used to examine this question. First, we used the PPARβ/δ antagonist GSK0660 [46]. Treatment of myotubes with 0.1 µM GSK0660 prevented the dexamethasone-induced increase in PPARβ/δ activity (Figure 4A), expression and activity of FOXO1 (Figure 4B and C) and expression of atrogin-1 and MuRF1 (Figure 4D and E). In addition, dexamethasone-induced increase in protein degradation (Figure 4F) and myotube atrophy (Figure 4G) were inhibited by GSK0660. Together, results shown in Figure 4 suggest that inhibition of PPARβ/δ activity with GSK0660 can prevent glucocorticoid-induced FOXO1 activation, upregulation of atrogin-1 and MuRF1, muscle proteolysis and atrophy.

### Downregulation of PPARβ/δ Expression with siRNA Reduces FOXO1 and MuRF1 Expression and Protein Degradation in Dexamethasone-treated Myotubes

**Figure 5:**
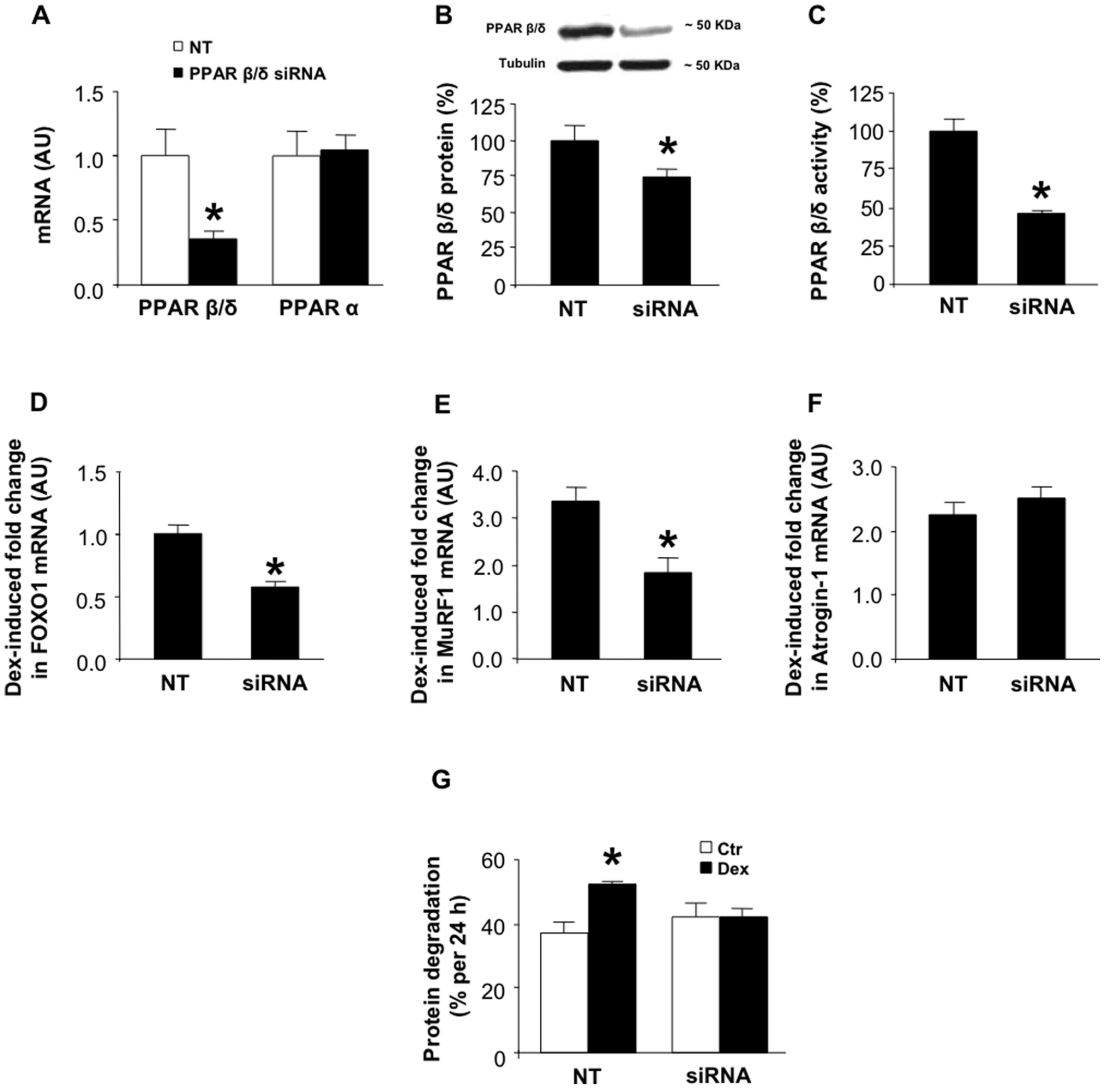
Downregulation of PPARβ/δ expression reduces FOXO1 and MuRF1 expression and protein degradation in dexamethasone-treated myotubes. *A*) mRNA levels for PPARβ/δ and PPARα in myotubes transfected with non-targeting (NT) or PPARβ/δ siRNA. *B*) PPARβ/δ protein levels in myotubes transfected with non-targeting (NT) or PPARβ/δ siRNA. Representative Western blots are shown in the upper and densitomtric quantifications in the lower panel. *C*) PPARβ/δ activity in myotubes transfected with non-targeting (NT) or PPARβ/δ siRNA. *D-F*) Dexamethasone-induced changes in FOXO1, MuRF1, and atrogin-1 mRNA in myotubes transfected with non-targeting (NT) or PPARβ/δ siRNA. *G*) Protein degradation in myotubes treated for 24 h with or without 1 µM dexamethasone following transfection with non-targeting (NT) or PPARβ/δ siRNA. Results are reported as arbitrary units (AU) or percent of control and are means ± SEM with n = 7 or 8/group. *p<0.05 vs appropriate control group by ANOVA (panel G) or Student’s t-test.

Although GSK0660 has been reported to be specific in its inhibitory effect on PPARβ/δ [46] non-specific effects of GSK0660 have been described as well, for example recruitment of various transcriptional corepressors [33]. In order to avoid the potential influence of non-specific effects of GSK0660, a second approach was used to test whether inhibition of PPARβ/δ may prevent dexamethasone-induced catabolic responses in cultured myotubes. In this experiment, the expression of PPARβ/δ was reduced by using PPARβ/δ siRNA. Transfecting myotubes with the siRNA construct resulted in an approximately 65% reduction of PPARβ/δ mRNA levels (Figure 5A). This effect of PPARβ/δ siRNA did not reflect a generalized reduction of gene transcription since the expression of another PPAR family member, PPARα, was not influenced by PPARβ/δ siRNA (Figure 5A). This was an important observation because both PPARα and PPARβ/δ are expressed in skeletal muscle and a significant redundancy in the regulatory effects of PPARα and PPARβ/δ has been reported [26]. PPARβ/δ protein levels and activity were reduced by approximately 30% and 60%, respectively, in PPARβ/δ siRNA-transfected myotubes (Figure 5B and C). Importantly, downregulation of PPARβ/δ expression with siRNA reduced FOXO1 and MuRF1 expression in dexamethasone-treated myotubes (Figure 5D and E). Surprisingly, PPARβ/δ siRNA did not influence atrogin-1 mRNA levels in dexamethasone-treated myotubes (Figure 5F). This was different from the results observed after treatment with GSK0660 which resulted in reduced expression of both atrogin-1 and MuRF1 in dexamethasone-treated myotubes (see Figure 4D and E). Although we do not have an explanation for these apparently contradictory results at present, the different effects of GSK0660 and PPARβ/δ siRNA with regards to atrogin-1 expression may reflect non-specific effects of GSK0660 or different degrees of PPARβ/δ inhibition by GSK0660 and PPARβ/δ siRNA. Importantly, however, downregulation of PPARβ/δ expression with siRNA blocked the dexamethasone-induced increase in protein degradation (Figure 5G), similar to the effect of GSK0660 (compare with Figure 4F).

### Treatment of Rats with GSK0660 Prevents Dexamethasone-induced Muscle Wasting

**Figure 6:**
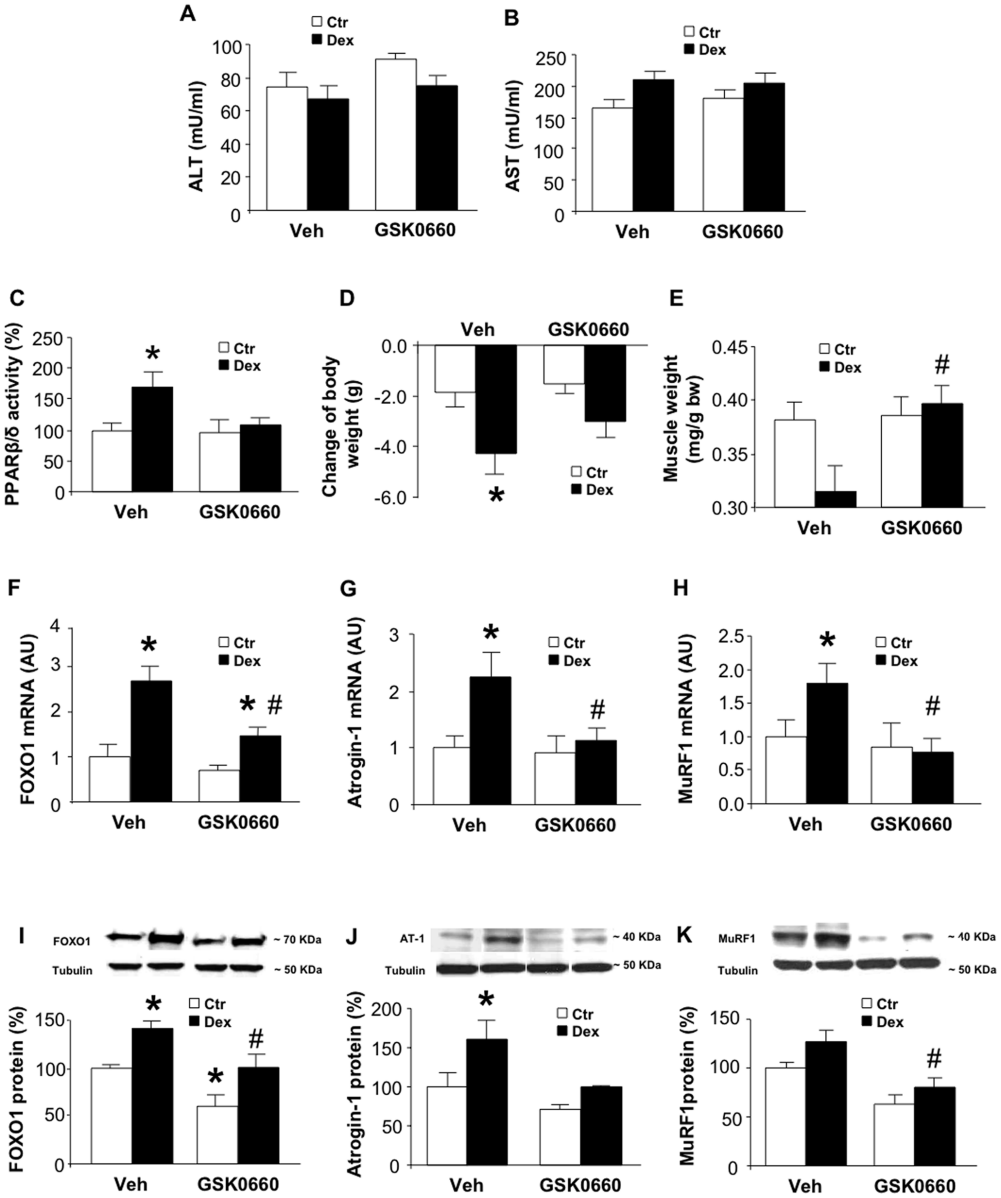
Treatment of rats with GSK0660 prevents dexamethasone-induced muscle wasting. *A)* and *B*) Serum ALT and AST levels in control and dexamethasone-treated (10 mg/kg) rats that were administered vehicle or GSK0660 (1 mg/kg). *C*) PPARβ/δ activity in EDL muscles from control and dexamethasone-treated rats that were administered vehicle or GSK0660. *D*) Change of body weight in control and dexamethasone-treated rats that were administered vehicle or GSK0660. *E*) Muscle weight in control and dexamethasone-treated rats that were administered vehicle or GSK0660. *F-H*) mRNA and *I-K*) protein levels for FOXO1, atrogin-1, and MuRF1 in muscles from control and dexamethasone-treated rats that were administered vehicle or GSK0660. Results are means ± SEM with n = 8/group. *<0.05 vs appropriate control group; #p<0.05 vs Veh/Dex group by ANOVA.

Although the results reported here provide important novel information regarding the role of PPARβ/δ in glucocorticoid-induced muscle atrophy and the effects of PPARβ/δ inhibition in dexamethasone-treated myotubes, it is important from a clinical standpoint to determine whether treatment with a PPARβ/δ inhibitor can prevent glucocorticoid-induced muscle wasting in vivo. In order to address this question, we administered GSK0660 to dexamethasone-treated rats. The dose of GSK0660 used here (1 mg/kg) was based on a previous report by Sanchez-Siles et al. [42]. The treatment was well tolerated as suggested by unchanged liver function tests (Fig. 6A and B) and the absence of mortality among GSK0660-treated rats. Treatment of rats with 10 mg/kg of dexamethasone resulted in an approximately 75% increase in PPARβ/δ activity in skeletal muscle and this effect of dexamethasone was abolished by GSK0660 (Figure 6C). The dose of dexamethasone used here was based on previous reports in which treatment of rats with 10 mg/kg of dexamethasone resulted in increased ubiquitin-proteasome-dependent muscle proteolysis and upregulated expression of FOXO1, atrogin-1, and MuRF1 [35]–[37], supporting the catabolic effects of glucocorticoids [2], [3]. Here we found that treatment with GSK0660 reduced the loss of body and muscle weight in dexamethasone-treated rats (Figure 6D and E) suggesting that GSK0660 can prevent glucocorticoid-induced muscle wasting in vivo. This was further supported by GSK0660-induced inhibition of FOXO1, atrogin-1 and MuRF1 mRNA and protein levels in dexamethasone-treated rats (Figure 6F–K). Together, results shown in Figure 6 suggest that glucocorticoids increase PPARβ/δ activity in skeletal muscle in vivo and that inhibition of PPARβ/δ activity can rescue muscle from the catabolic effects of glucocorticoids.

**Figure 7:**
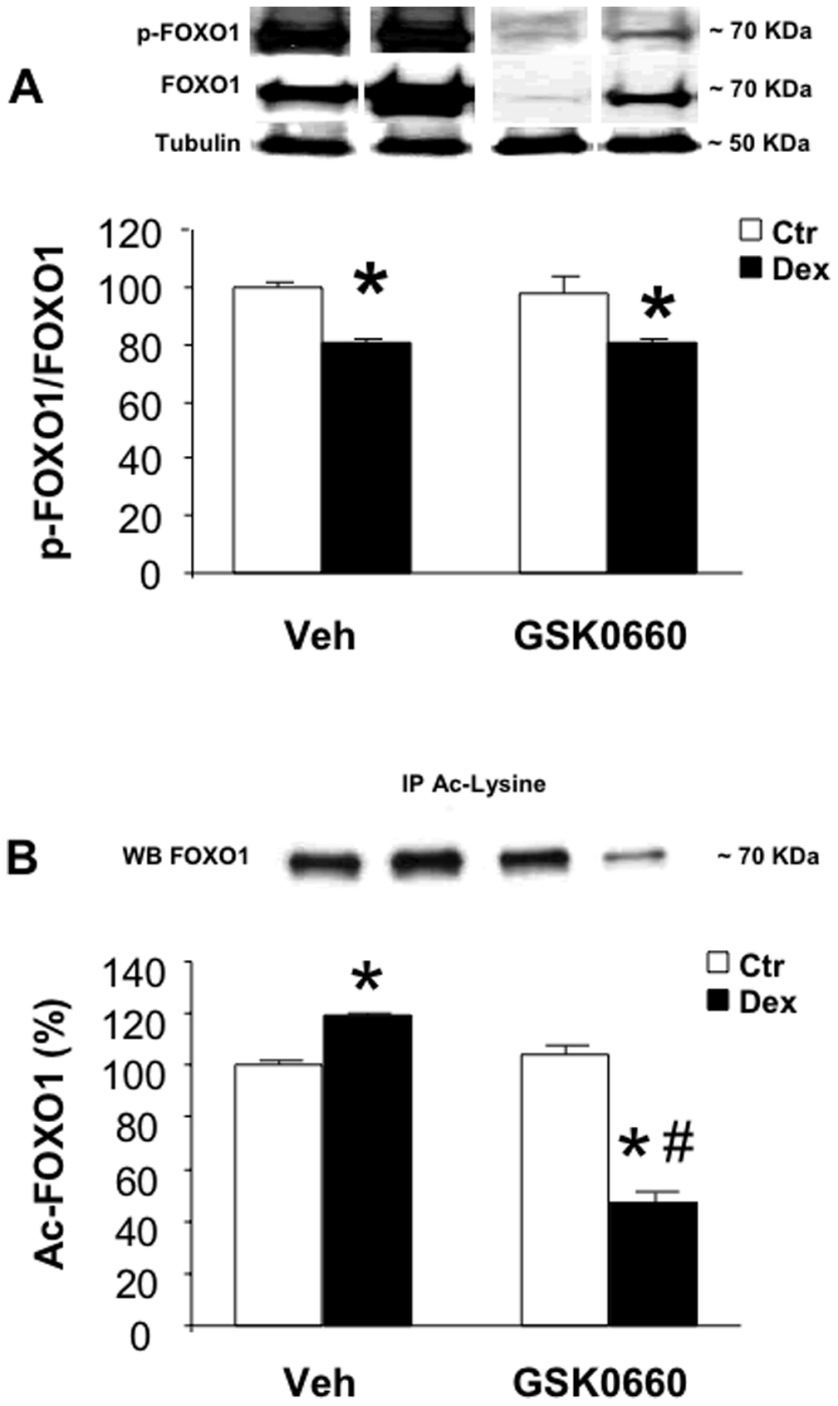
Treatment of rats with GSK0660 prevents dexamethasone-induced increase in total FOXO1, p-FOXO1, and Ac-FOXO1. *A)* p-FOXO1 and total FOXO1 levels in EDL muscles from control and dexamethasone-treated rats that were administered vehicle or GSK0660. *B)* Ac-FOXO1 levels in EDL muscles from the same groups of rats that were studied in panel A. Results are means ± SEM with n = 6–8/group. *p<0.05 vs Ctr; #p<0.05 vs corresponding vehicle group by ANOVA.

Although our experiments in cultured myotubes provided evidence that increased FOXO1 activity following GW0742-induced activation of PPARβ/δ was associated with increased total FOXO1 levels and reduced p-FOXO1/FOXO1 ratio (see Figure 1D), it is not known if the same mechanism regulates dexamethasone-induced FOXO1 activation in vivo and if PPARβ/δ is involved. To examine that question, we determined total FOXO1 and p-FOXO1 levels in EDL muscles of rats treated with dexamethasone and GSK0660. Treatment of rats with 10 mg/kg of dexamethasone resulted in increased muscle levels of both total and phosphorylated FOXO1 with the increase in total FOXO1 being greater than the increase in p-FOXO1, resulting in a reduced p-FOXO1/FOXO1 ratio (Figure 7A). When rats were treated with GSK0660, total FOXO1 and p-FOXO1 levels were reduced in both control and dexamethasone-treated rats. Taken together, these observations suggest that dexamethasone-induced FOXO1 activation in vivo is associated with increased total FOXO1 levels (similar to dexamethasone-induced activation in cultured myotubes) and that FOXO1 levels are regulated by PPARβ/δ activity in both control and dexamethasone-treated rats.

In recent experiments, we found that treatment of cultured myotubes with dexamethasone resulted in increased levels of acetylated FOXO1 [44]. Here, we found that treatment of rats in vivo with dexamethasone also increased muscle levels of Ac-FOXO1 (Fig. 7B). Treatment of rats with GSK0660 alone did not influence muscle levels of Ac-FOXO1. In contrast, GSK0660 did not only prevent the dexamethasone-induced increase in Ac-FOXO1 levels but reduced the levels below control levels.

### Sepsis-induced Muscle Wasting in Rats is Improved by Treatment with GSK0660

**Figure 8:**
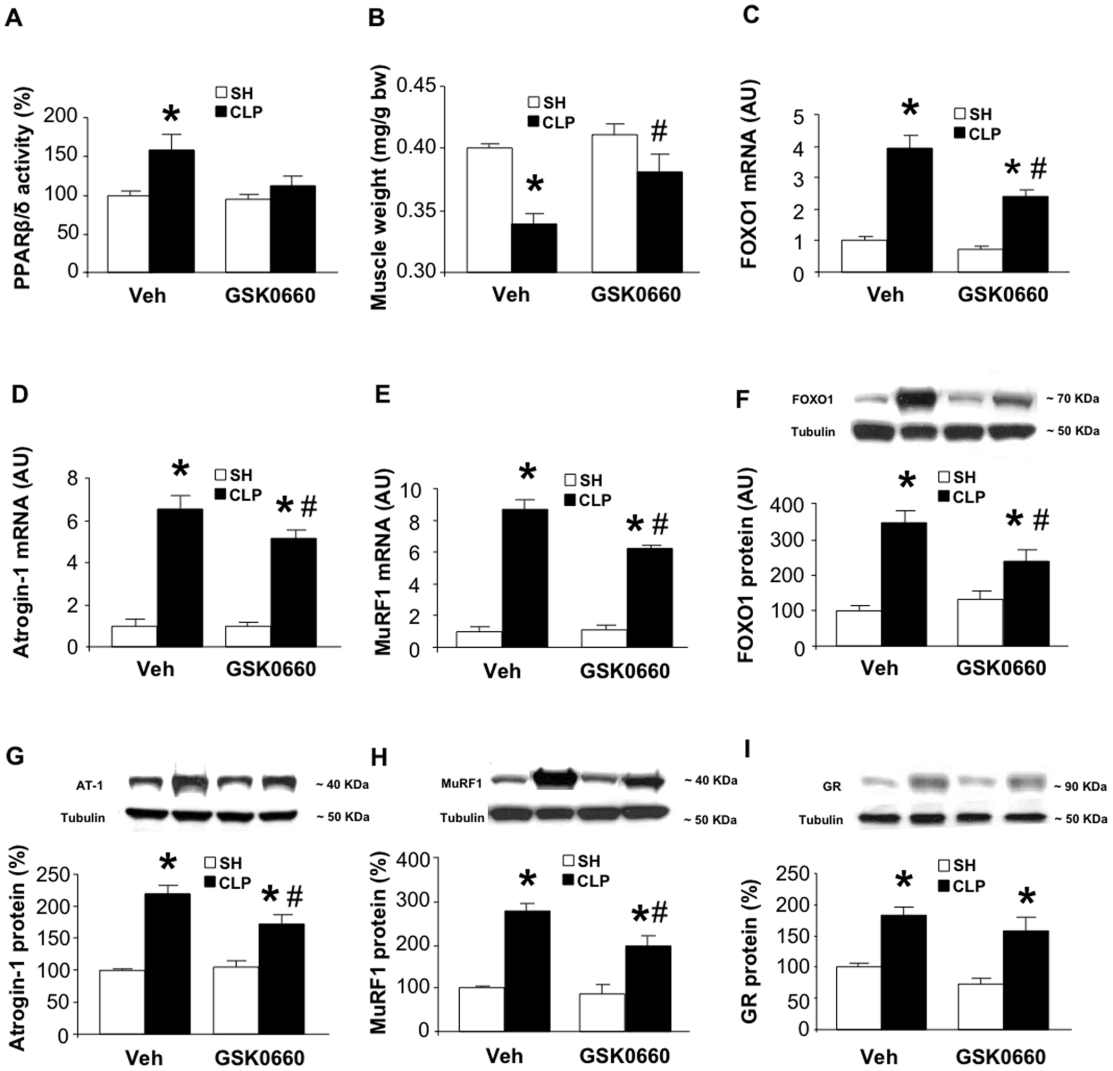
Sepsis-induced muscle wasting in rats is improved by treatment with GSK0660. *A*) PPARβ/δ activity in EDL muscles from sham-operated (SH) and septic (CLP) rats treated with vehicle or GSK0660 (1 mg/kg). *B*) Muscle weight in sham-operated and septic rats treated with vehicle or GSK0660. *C-E*) mRNA and *F-H*) protein levels for FOXO1, atrogin-1, and MuRF1 in muscles from sham-operated and septic rats treated with vehicle or GSK0660. *I)* GR protein levels in EDL muscles from sham-operated and septic rats treated with vehicle or GSK0660. Results are means ± SEM with n = 8/group except in panel I where n = 4/group. *p<0.05 vs appropriate sham group; #p<0.05 vs Veh/CLP by ANOVA.

The effects of dexamethasone in cultured myotubes and in rats were examined in the present study because high levels of glucocorticoids by themselves induce muscle wasting and because muscle wasting in several catabolic conditions, including sepsis, is mediated by glucocorticoids [2], [3], [35], [38], [39]. Several previous reports provided evidence that sepsis is associated with loss of muscle mass and upregulated expression and activity of FOXO1, atrogin-1 and MuRF1 [12], [36], [37], [39], [56]. In contrast, the influence of sepsis on muscle PPARβ/δ activity in skeletal muscle has not been reported and it is not known if treatment with a PPARβ/δ inhibitor can prevent sepsis-induced muscle wasting. Here, we found that induction of sepsis by cecal ligation and puncture (CLP) in rats resulted in an approximately 50% increase in PPARβ/δ activity in skeletal muscle and this effect of sepsis was blocked by treatment with GSK0660 (Figure 8A). Importantly, treatment with GSK0660 reduced sepsis-induced loss of muscle mass (Figure 8B) and blunted (but did not completely prevent) sepsis-induced upregulation of FOXO1, atrogin-1 and MuRF1 mRNA (Figure 8C–E) and protein levels (Figure 8F–H). Taken together, these results suggest that sepsis-induced muscle wasting is associated with increased PPARβ/δ activity and can be improved by treatment with a PPARβ/δ inhibitor.

In previous studies, sepsis in rats was associated with increased expression of the GR in skeletal muscle [57] and sepsis-induced muscle wasting as well as expression of atrogin-1, MuRF1, and FOXO1 were prevented by treatment with the glucocorticoid receptor antagonist RU38486 [12], [35], [38], [39]. In the present study, the dexamethasone-induced PPARβ/δ activation in cultured myotubes was GR-dependent (see Figure 3C). Taken together, these observations raise the possibility that the effects of GSK0660 in septic rats may reflect prevention of the sepsis-induced increase in muscle GR levels. To test that possibility, we determined GR protein levels in EDL muscles from septic rats treated with GSK0660. As expected [57], GR levels were increased in muscle from septic rats but this increase was not prevented by GSK0660 (Figure 8I). Therefore, if sepsis-induced PPARβ/δ activation is GR-dependent (as suggested by the results in dexamethasone-treated myotubes, see Figure 3C), it may reflect other mechanisms than increased GR abundance, for example an increased interaction between the GR and PPARβ/δ as suggested by results in dexamethasone-treated myotubes (See Figure 3D).

## Discussion

The present results suggest that the transcription factor PPARβ/δ regulates FOXO1 expression in skeletal muscle and plays an important role in glucocorticoid- and sepsis-induced muscle wasting. In addition, results suggest that inhibition of PPARβ/δ activity can prevent the catabolic effects in skeletal muscle caused by sepsis and high glucocorticoid levels. The study is important because it provides novel information about mechanisms involved in muscle wasting and suggests, for the first time, that PPARβ/δ may be a target for prevention of sepsis- and glucocorticoid-induced muscle wasting. The observations have significant clinical implications because loss of muscle mass has multiple severe consequences, including muscle weakness, delayed ambulation with increased risk for thromboembolic complications in critical illness, and risk for pulmonary complications and difficulties weaning patients from mechanical ventilatory support when respiratory muscles are affected [58]–[61].

The study by Nahle et al. [20] suggests that PPARβ/δ activation in skeletal muscle is at least in part regulated by increased expression and activity of the fatty acid translocase CD36 and elevated fatty acid levels. Although those mechanisms were not investigated in the present report, studies from other laboratories suggest that the expression and activity of CD36 and other fatty acid transport proteins (FATPs) as well as fatty acid accumulation are increased in catabolic muscle [62]–[65]. In recent experiments in our laboratory, CD36 expression was increased in skeletal muscle during sepsis and treatment of cultured myotubes with dexamethasone resulted in increased expression of CD36 and FATP1 (unpublished observations). Thus, it is likely that increased fatty acid transport is involved in PPARβ/δ activation during sepsis and glucocorticoid treatment. Importantly, the present study provides evidence for an additional, novel, mechanism of PPARβ/δ activation by showing that glucocorticoids and the GR are involved in PPARβ/δ activation in skeletal muscle and that the role of the GR may reflect the formation of a complex between the GR and PPARβ/δ. Because sepsis-induced muscle wasting is regulated by glucocorticoids and can be prevented by the GR antagonist RU38486 [35], [38], [39], it is possible that glucocorticoids and the GR participate in the activation of PPARβ/δ during sepsis as well. Our results in GSK0660-treated septic rats suggest that the role of the GR in sepsis-induced PPARβ/δ activation does not depend on the increased levels of the GR in septic muscle but may reflect other mechanisms, such as an increased protein-protein interaction between the GR and PPARβ/δ as suggested by results in dexamethasone-treated myotubes. It should be noted that although the present study and a report by Nie et al. [54] suggest that a physical interaction between the GR and PPAR transcription factors may be a mechanism of glucocorticoid-induced PPAR activation, other mechanisms may also be involved, such as interaction with nuclear coactivators shared by PPAR transcription factors and the GR [66]–[68].

Even though evidence for PPARβ/δ-regulated FOXO1 expression in cultured muscle cells was reported previously [20], the downstream consequences of PPARβ/δ activation examined in that report were changes in PDK4 expression and activity and glucose oxidation. The present study provides the first evidence of a PPARβ/δ-FOXO1-atrogin-1/MuRF1 pathway in glucocorticoid- and sepsis-induced muscle wasting. Our observations confirm the central role of FOXO1 in muscle wasting [8]–[13], and suggest that inhibition of PPARβ/δ may prevent loss of muscle mass in catabolic conditions secondary to inhibition of FOXO1 expression and activity. The fact that FOXO1, as well as FOXO3a, regulate not only genes in the ubiquitin-proteasome proteolytic pathway, such as atrogin-1 and MuRF1 [11], [13], but also genes in the autophagy-lysosomal pathway [18], [19], makes PPARβ/δ an even more attractive target for prevention of muscle wasting.

Although reduced phosphorylation is an important mechanism of FOXO1 activation [47], the present results and previous studies [8], [12] suggest that increased levels of total FOXO1, even in the presence of increased p-FOXO1 levels, may result in a net increase in unphosphorylated (activated) FOXO1 as suggested by a reduced p-FOXO1/FOXO1 ratio. Of note, the present results suggesting that PPARβ/δ-induced FOXO1 activation was associated with increased abundance of total FOXO1 without reduction of p-FOXO1 levels do not rule out the possibility that other posttranslational modifications were involved in the increased FOXO1 activity. For example, studies suggest that increased acetylation results in increased FOXO1 activity [48], [49] and in recent studies we found evidence that increased FOXO1 acetylation is involved in the catabolic response to dexamethasone in cultured myotubes [44], [50]. Results in the present study suggest that PPARβ/δ activation is not sufficient to increase FOXO1 acetylation (as illustrated in GW0742-treated myotubes). In contrast, PPARβ/δ may be involved in glucocorticoid-induced FOXO1 acetylation as suggested by results from dexamethasone-treated rats that were administered GSK0660.

The present observation that treatment with GSK0660 reduced, but did not completely prevent, the increase in FOXO1, atrogin-1, and MuRF1 expression in septic rats, despite normalized PPARβ/δ activity, probably reflects the influence of additional transcription factors involved in muscle wasting, including NF-kB [69]–[71] and C/EBPβ [30], [72]. Prevention of muscle wasting during sepsis and other catabolic conditions will probably require a multifaceted approach addressing multiple mechanisms involved in loss of muscle mass. The present study is important because it provides the first evidence that inhibition of PPARβ/δ activity may be an essential component of treatment aimed at rescuing muscle from the influence of catabolic conditions.

The present results are apparently contradicted by previous studies suggesting that PPARβ/δ activation may be protective, rather than harmful, in skeletal muscle, for example by increasing insulin sensitivity [73], [74] and by enhancing mitochondrial biogenesis and function [75]–[77]. Those observations are pertinent for the present study because previous reports suggest that sepsis- and glucocorticoid-induced muscle wasting is associated with insulin resistance [78], [79] and reduced mitochondrial biogenesis and function [80], [81]. Of note, however, some of the potentially protective effects of PPARβ/δ are controversial. Thus, evidence of unaffected [82]–[84] or even reduced insulin sensitivity [85] following activation of PPARβ/δ was reported recently. In other studies, treatment with a PPARβ/δ agonist either did not influence mitochondrial function [27] or resulted in uncoupled oxidative phosphorylation in isolated muscle mitochondria [86], changes that are typically associated with increased generation of oxygen radicals and deleterious effects on mitochondrial function [87]. Regardless of the influence of PPARβ/δ on other metabolic changes accompanying muscle wasting, the present results provide strong evidence that activation of PPARβ/δ results in upregulation of genes in the ubiquitin-proteasome pathway, stimulation of protein degradation, and atrophy of muscle cells and, importantly, that inhibition of PPARβ/δ improves glucocorticoid- and sepsis-induced muscle wasting.
